# Adoption of Pharmacogenomic Testing: A Marketing Perspective

**DOI:** 10.3389/fphar.2021.724311

**Published:** 2021-09-17

**Authors:** Margarita-Ioanna Koufaki, Kariofyllis Karamperis, Polixeni Vitsa, Konstantinos Vasileiou, George P. Patrinos, Christina Mitropoulou

**Affiliations:** ^1^ University of Patras School of Health Sciences, Department of Pharmacy, Laboratory of Pharmacogenomics and Individualized Therapy, Patras, Greece; ^2^ The Golden Helix Foundation, London, United Kingdom; ^3^ United Arab Emirates University, College of Medicine and Health Sciences, Department of Pathology, Al-Ain, United Arab Emirates; ^4^ United Arab Emirates University, Zayed Center for Health Sciences, Al-Ain, United Arab Emirates

**Keywords:** personalized medicine, pharmacogenomics, marketing strategies, innovation, awareness, genetic testing services, patients

## Abstract

Pharmacogenomics is becoming an important part of clinical practice and it is considered one of the basic pillars of personalised medicine. However, the rate of pharmacogenomics adoption is still low in many healthcare systems, especially in low- or middle-income countries. The low level of awareness of healthcare specialists could be a potential reason due to which pharmacogenomics application is still in a premature stage but there are several other barriers that impede the aforementioned process, including the lack of the proper promotion of pharmacogenomic testing among interested stakeholders, such as healthcare professionals and biomedical scientists. In this study, we outline the available marketing theories and innovation that are applied to personalized medicine interventions that would catalyze the adoption of pharmacogenomic testing services in clinical practice. We also present the current ethical and legal framework about genomic data and propose ways to tackle the main concerns mentioned in the literature and to improve the marketing perspective of PGx.

## Introduction

Pharmacogenomics (PGx) is an important component of the various clinical actionable omics disciplines and it is considered one of the basic pillars of personalized medicine. The completion of the Human Genome Project in 2003 along with the presentation of the Precision Medicine (now AllOfUs; https://allofus.nih.gov) initiative in 2015 (Mullard, 2015), boosted PGx recognition and therefore it became known for its potential applications and benefits in patient care, while other national-wide genomic medicine initiatives, such as the Genomics England are currently underway (reviewed in Patrinos et al., 2020). The implementation of PGx significantly contributes to the decrease of health care cost owing to several factors such as the drop of incidence of adverse drug reactions, the adjustment of drug dosing, the possibility of performing less unsuccessful clinical trials, and in shorten time, the adoption of a personalized therapeutic approach based on a patient genetic makeup (Yau and Haque, 2016; Klein et al., 2017). All these positive aspects drew public’s attention and gained unexpected popularity in many countries such as the example of Singapore and the launch of a 10-years program of Precision Health Research (https://www.npm.sg), resulting in its wider application in the clinical setting.

However, apart from the significant technological advances in the genomics technologies and knowledge, the rate of adoption of personalized medicine interventions and in particular of PGx remains low in many healthcare systems, underlying that there are certain barriers impeding their application. This is not unforeseen as the implementation of PGx and personalized medicine interventions is the synergistic outcome of several parameters, including mostly those that touch upon public health genomics disciplines and unfortunately nowadays remain poorly addressed. Particularly, one of the most important hurdles that hold back the broader clinical implementation of PGx testing services is their widespread provision and availability to the patients and the general public. To this end, the establishment of adequate promotion channels for the PGx testing services, targeting differently healthcare professionals and patients aligned to their genetic knowledge and awareness, is of utmost importance to maximize their benefit for the patients and society.

In this paper, we sought to outline the existing level of pharmacogenomics awareness of healthcare professionals and general public and to propose how existing marketing theories can be applied to catalyze the adoption of pharmacogenomic testing and personalized medicine interventions in clinical practice. We also strive to propose ways to tackle the main concerns mentioned in the literature.

## Applying the Marketing Mix in PGx and Personalized Medicine

We need first to define the product's and/or service's characteristics, the customers' features and the market that the product and/or service is addressed to. In general, the focus of marketing has changed and it is more customer-oriented than product-oriented. Knowing your customer’s needs at a certain point will define whether a product can be considered as a valuable addition to public health. Moreover, and in order to introduce a marketing strategy, marketers must approach the customer in a context that takes into consideration several factors. The market’s competition, the governmental policies and regulations, combined with the broader economic, social and political macroeconomic forces shape the evolution of markets and determine the marketing strategies. Therefore, the task of marketing, known as customer-perceived value, is to create a customer value greater than other competitors, in terms of benefits and costs.

Marketing as a scientific field involves complicated processes other than simply advertising or selling a product. Its main goal is to develop and manage a product/service that will satisfy specific needs by making it available at the right place, at the right time, at the price that is acceptable to customers, and with the right people. Even if the current trends are different, the objective is still the same; to make a profit. However, the means of achieving this objective have expanded to include the entire marketing mix or the “4Ps” as they became known, namely product, price, promotion, and place.

In order to better comprehend the special features of PGx testing and personalized medicine interventions, we will base our analysis on the 4Ps context, as defined below:

• Product: In the case of PGx, products are defined as the concept of PGx and personalized medicine interventions and applications. Once the product is launched into the market, it goes through four stages: import, growth, maturation, and decline, also known as a product life cycle. At the import stage, the product is advertised to become known to the public and available on the market. At this phase, the product is unlikely to be profitable, therefore, it should be monitored continuously until there is an increase in sales. In case that the product isn’t profitable, the safest alternative would be to withdraw it from the market. As soon as the product sales begin to rise, it enters the growth phase, which is characterized by increased sales and profits. The next stage is called maturation stage and it is important for products already on the market since the competition becomes stronger. At this point, the producer should develop new ideas and invest money in product research and development aiming to enter a new product in the production pipeline. Finally, during the decline stage, as product’s profits are no longer high, it may be withdrawn from the market. Genetic tests and personalized medicine interventions in general, particularly those related to PGx or to genetic predisposition to an inherited disease, are considered to belong to the first or second stage of development for almost every country.
• Price: The price range of a PGx testing service depends on the country, the reimbursement policy, and the technology. For example, whole-genome and/or whole-exome sequencing, or targeted resequencing, always tightly coupled to data interpretation, are emerging and very expensive technologies. The cost and the price of a genetic test plays a crucial role in the widespread application of personalized medicine interventions. Owing to the fact, that health is a key priority for most people, the price of testing must be affordable and/or be reimbursed by the government and used in multiple patient groups.
• Place: It refers to the way the product is distributed to the customer. PGx testing service is commonly provided via, ideally accredited, laboratories and public or private hospitals. In this market, direct distribution channels also known as Direct-to-Consumer (DTC) approaches, in which products are sold directly from the producer to the end-users (patients and general public) are usually avoided because the results of a genetic test have to be interpreted by an expert such as a specialized doctor and/or geneticist. In addition, the prevalence of genetic disorders varies significantly among different populations, and as such PGx tests must be provided according to the needs of each population.
• Promotion: Based on the targeted customer group (individual, organization, academia, or pharmaceutical industry) promotional strategies and methods may be slightly different. The basic elements of the standard promotional strategy are advertising, personal selling, publicity and public relations, sponsorship, direct mail, and sales promotions. Advertising is general communication aimed at a relatively large and diverse target audience, whereas personal selling involves more specific communications to one person or specific groups. The marketers invest budget and effort in mobile marketing, behavioral digital marketing, and social media marketing. In the latter case, even though this promotional approach has been developed very recently, it has shown great potential.

As previously mentioned, the target audience for genetic testing includes physicians, healthcare professionals, and patients. For those groups, different approaches must be adopted and followed. As far as physicians are concerned, it is important to adequately inform them regarding the benefits of using genetic tests for the early diagnosis of hereditary diseases and for a more rationalized drug use and prescription that can minimize the risk for a patient to develop adverse drug reactions. Similarly, patients should be aware of the benefits of personalized therapeutic schemes and of undertaking a genetic test because a person’s genetic profile constitutes a valuable source of information about his/her health outcomes. Moreover, being able to diagnose a genetic disease at an early stage will enhance the role of prevention, avoid future treatment costs, and, most importantly, improve people’s quality of life.

In contrast to the majority of consumer products, genetic testing like PGx requires specific and scientifically sound promotional means such as scientific journals and websites that can appeal to healthcare professionals interest as well as informational days for physicians to raise awareness about the usefulness and relevance of these tests for their patients. The commonly used direct marketing techniques of genetic tests to the general public, like advertisements in television and newspapers, cold calls, or mass postal or e-mail, are considered to be inappropriate for such a specialized type of service. Prior to the promotion of a genetic test, it is essential to recruit well-trained biomedical scientists as marketing staff, to provide customers with all necessary information and guidance in a proper and scientifically acceptable manner.

## SWOT Analysis

In the previous paragraph, we focused on the “4P” principle that aims to define and analyze the product and/or service and its characteristics. SWOT analysis is an official tool that helps towards the identification of opportunities and threats of a market overall, and in our case that of the PGx healthcare provider.

**FIGURE 1:**
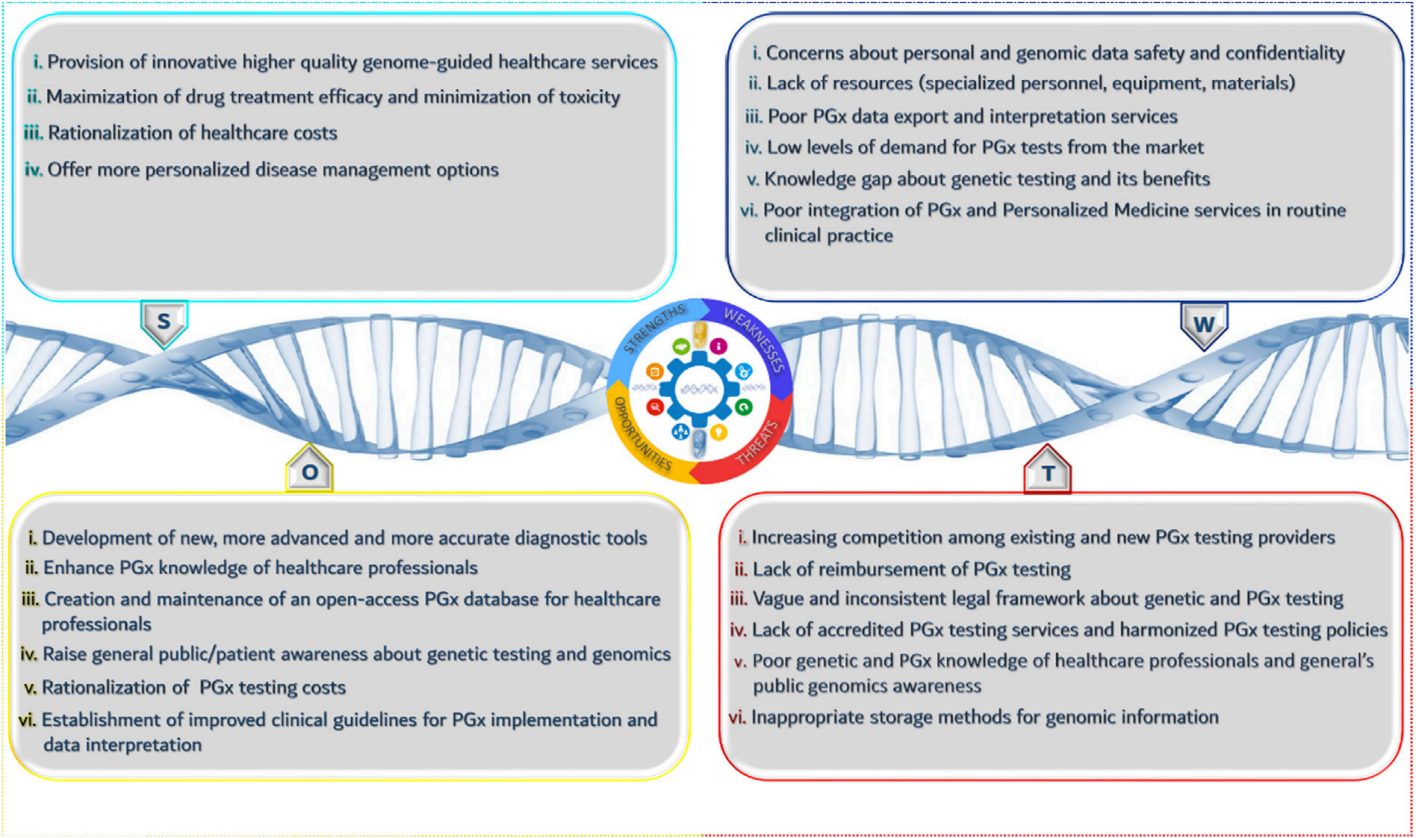
SWOT analysis for the implementation of PGx services by healthcare providers.

According to Figure 1, there are several strengths and opportunities for the wider adoption of PGx testing along with weaknesses and threats. The positive impact of PGx in clinical practice in terms of cost savings and quality of life improvement outweighs the existing drawbacks related to the low level of public’s knowledge about genetics and their ethical concerns. Beyond doubt, introducing and establishing PGx in healthcare will bring a series of different advancements and improvements that will increase the role of PGx research, reduce the cost for genetic services, ameliorate governmental health policies, and sensitize healthcare professionals and patients about PGx importance and benefits. Moreover, the innovative character of PGx will spark stakeholders interest to invest in such kind of services and it will give a boost to the development of new complementary services and applications to better support and facilitate PGx clinical implementation.

Nonetheless, the presence of specific threats may hamper the rapid progression of PGx and personalized medicine interventions. The lack of accreditation systems such as ISO or CLIA-accreditation focused on PGx testing, to ensure the quality and reproducibility of genetic results can raise several questions and doubts about the efficacy of these tests, having a negative impact on PGx adoption by healthcare professionals. In particular, their reluctance of adopting genome-guided therapeutic interventions can partly be justified due to the lack of PGx reimbursement and the absence of well-established and interoperable electronic health records in many countries. Another important aspect is the storage of raw data derived from genetic testing. Living in the era of big data, data storage technologies and equipment remain a common bottleneck that scientists need to resolve, as a simple genetic test can generate a large amount of raw data.

Aside from data storage, many people are also worried about the use of those data in terms of data protection and privacy because, there is a vague and unspecific legal framework. As it happens with every innovation, PGx and personalized medicine interventions need to be promoted under a very strict legal framework, because they touch upon sensitive personal data.

On the one hand, in Europe, in line with the previous legal framework (Directive 95/46/EC) the new EU General Data Protection Regulation (GDPR) 2016/679 defines genetic data as “personal data, namely information concerning an identified or identifiable natural person (“data subject”), whose identity can be verified, directly or indirectly, in particular by reference to an identifier, such as, inter alia, the factors that characterize the genetic identity of the natural person (Article 4)”. For the first time, there is an explicit definition of genetic data described as “personal data relating to the genetic characteristics of a person inherited or acquired, as derived, in particular, from an analysis of a biological sample of that natural person and which provide unique physiological information or the health of that natural person (Article 4)”.

On the other hand, in the United States, there is a relevant law named as the Genetic Information Nondiscrimination Act (GINA) 2008, which is an Act of Congress designed to prevent and prohibit cases of genetic discrimination. GINA does not allow health insurers to discriminate or even exclude enrollees based on their hereditary data. More precisely, health insurers should not utilize hereditary data to make qualification, inclusion, endorsing, or premium-setting choices. In addition, health insurers may not ask for or require people or their relatives to undergo a genetic testing or to share their hereditary data. As pinpointed in the law, hereditary data include family medicinal history, sickness reports in close relatives or other information regarding people’s genetic tests. There are similar legal provisions in countries across the world as outlined in Kordou et al. (2020) but there isn’t a global directive.

To conclude with, evidently a strict and coherent legal framework should be considered, in principle, to provide the guarantees for the effective protection of personal genetic data. However, it remains to see how the public authorities implement the aforementioned framework and how the court will interpret it in practice.

## Diffusion of Personalised Medicine as an Innovative Product

Previously, we have identified the market and its characteristics. In this paragraph, we propose ways towards adoption of the innovative PGx testing services from the healthcare providers and means of diffusion of personalised medicine interventions. Innovation is not simply an idea but rather a process of designing and developing an innovative product and/or service with the aim to be launched successfully into the market. As such, the adoption and particularly the diffusion of all innovative products and services is an issue of great importance. This is also the case for almost all healthcare innovations, including PGx testing and those of Personalized Medicine overall, being a recent trend in the healthcare industry that ameliorates healthcare outcomes. Even though achieving optimal healthcare is thought to be one of the most significant goods in all societies and there are highly specialized professionals, i.e., health professionals, consultants, and experts, responsible for the decision-making about innovation adoption and diffusion, its diffusion rate remains low. Several research models have been employed to investigate the process of healthcare innovations diffusion, with various types of Technology Acceptance Model (TAM) and Diffusion of Innovation theory (DOI) being the predominant (Greenhalgh et al., 2005; Ha et al., 2018; Jacob et al., 2020). The DOI developed by Everett Rogers (2003) provides a conceptual framework that explains/describes, inter alia, the individual innovation-decision process (e.g. physicians, pharmacists, patients).

The individual innovation-decision process portrays the path that stakeholders follow to adopt or reject an innovation. It consists of five steps: 1) knowledge, 2) persuasion, 3) decision, 4) implementation, and 5) confirmation. In the case of PGx and Personalized Medicine, an individual is informed about the innovative product via various communication channels and searches for more information about it. The level of the stakeholders knowledge is affected, inter alia, by specific factors, classified as socioeconomic characteristics, personality variables, and communication behavior. Based on their knowledge, individuals, like general practitioners, medical specialists, genetic counselors, and clinical geneticists, proceed to the next step, that of persuasion. In this stage, they form their attitudes, whether favorable or unfavorable, towards the Personalized Medicine innovation, facilitated by their interactions with their colleagues and peers. The developers and advocates of PGx and Personalized Medicine innovations should always keep in mind that individuals attitudes towards innovation rely on their perceived features, namely competitive advantage, compatibility (concerning individual’s needs and experiences), complexity (affecting its ease of use), trialability (permitting to experiment on a trial basis) and observability (readily comprehensible results). The persuasion stage is followed by the decision, in which the individuals decide whether to adopt or not the innovation. Adopting such as product or service and how quickly this will occur are positively associated with its trialability by a person.

The implementation stage marks the beginning of the innovation use. At the stage of confirmation, the individual makes his/her final decision whether to continue or discontinue the use of the innovation and, in case of initial rejection, if he/she will finally adopt it or keep rejecting it forever. This decision is based on further information acquired in the pursuit of supporting his/her decision, and in case of gathering conflicting information, the adoption may be reversed. Rogers (2003) identified five types of variables that determine an innovation’s rate of adoption:

a. the perceived attributes of innovation mentioned above,
b. the type of innovation-decision (optional, collective, authority),
c. the type of communication channels, namely those employed for the innovation diffusion at various stages in the innovation-decision process,
d. the model of the social system in which the innovation is being diffused (e.g. norms, degree of network interconnectedness), and
e. the extent of change agents’ promotion efforts in diffusing the innovation.

## Discussion

Based on the literature, there are many international publications focused on the level of awareness of pharmacists, physicians, and other healthcare professionals being at different levels from undergraduate students to professionals (Mai et al., 2014; Rahma et al., 2020a; Rahma et al., 2020b). These studies indicated that the level of awareness varied among the different countries, while an individual’s profession seems to decisively affect a person’s perception and attitude towards the role of PGx and its applications (Bonter et al., 2011).

In Egypt, according to Nagy and coworkers (2020), both pharmacists and physicians indicated that they obtained a relatively low level of PGx awareness but a rather positive attitude towards the clinical implementation of PGx testing. Also, Karuna et al. (2020) indicated similar results in Thailand in which 46.3% of the respondents had a poor knowledge, while the majority of those hadn’t recommended or prescribed a PGx test to patients in the past year. In contrast, in Jordan, 73.4% of physicians who participated in the Jarrar et al. (2019) study, knew about PGx and pinpointed that they had applied or used PGx in their clinical practice.

The level of awareness isn’t the only variable that have an impact in specialists perception about PGx, but individual’s attitude also plays an important role in the promotion and widespread implementation of PGx and Personalized Medicine interventions. According to Sindi et al. (2017), 80.5% of medical students inquired indicated that they were willing to take a genetic test to find out “what disease they might get in the future”, even if they characterized their knowledge about Personalised Medicine to be poor, while Abdela et al. (2017) indicated that the majority of respondents expressed a positive viewpoint about implementing a PGx testing to improve patient experience and receiving a better or additional training on the corresponding topic. Furthermore, many physicians claimed that PGx testing should shift its efforts and focus more on pharmacy practice as pharmacists are proved to have a better level of knowledge in the field (Giri et al., 2018; Algahtani, 2020). Overall, physicians are less informed about PGx and they don’t feel comfortable to interpret genetic data, whereas pharmacists appeared to have a better level of knowledge and understanding of PGx and they are more confident with its application (Elewa et al., 2015; Kudzi et al., 2015; Golbach, 2018).

The low level of awareness of healthcare specialists could be a potential reason due to which PGx adoption is still in a premature stage, however, several other barriers impede the aforementioned process. According to Rahma et al. (2020a), healthcare professionals lack adequate training and education on PGx and they are seriously concerned about the absence of evidence-based clinical guidelines. Lack of available resources such as laboratory equipment, infrastructure, and specialized personnel also constitutes an important factor for the low integration level of PGx, especially in less developed economies (Kudzi et al., 2015; Muzoriana et al., 2017). Moreover, PGx tests aren’t reimbursed or covered from medical insurance schemes in many countries (Patrinos & Mitropoulou, 2017), while Murphy et al. (2010) and Kricka et al. (2011) implied that there is “an inconsistent regulatory landscape” in the United States and Europe correspondingly.

Both of these aspects have a great impact on public opinion and perception of PGx (Kobayashi et al., 2011). Indeed, patients are worried about the protection of their personal information, and they believe that confidentiality and discrimination issues will be raised due to PGx (Haga et al., 2012; Rahma et al., 2020a). Finally, in many cases both healthcare specialists and patients highlighted that they weren’t willing to apply PGx testing owing to ethical, cultural, and religious matters (Rahma et al., 2020b).

## Conclusions and Future Perspectives

From the above, it is obvious that there are indeed various barriers that impact the adoption rate and promotion of PGx and personalized medicine interventions in clinical practice and it is urgent to improvise solutions to overcome them.

First of all, it is significantly important to promote and encourage genetics education and training of healthcare professionals, and especially physicians and pharmacists, concerning the role of PGx and personalized medicine interventions in the clinical setting and its applications. To do so, establishing appropriate PGx courses and modules in universities and colleges even at the undergraduate level along with setting up online training programs will enhance the level of understanding of corresponding specialists and increase the adoption rate of PGx (Kampourakis et al., 2014; Algahtani, 2020). In addition, the establishment of new undergraduate or postgraduate programs for clinical PGx and Genomic and Personalized Medicine is another potential solution to overcome the shortage in trained personnel.

Furthermore, according to the literature, many clinicians have raised the concern that they don’t have access to clinical guidelines and that the available support systems aren’t user-friendly. Admittedly, there is a series of available guidelines on the web but there is a lack of an organized repository dedicated to PGx, while these guidelines present a great heterogeneity even if they derived from the same evidence base (Koutsilieri and Patrinos, 2020; Nagy et al., 2020). Creating an open-access database to share and exchange information, under the auspices of major regulatory bodies, such as the United States Food and Drug Administration (FDA; www.fda.gov) and the European Medicines Agency (www.ema.europa.eu), would be beneficial and facilitate professional work while it can increase the existing knowledge about clinical utility. This would also include incorporating several PGx biomarkers, that have already been identified with clinical utility, into drug labels (Klein et al., 2017). Highlighting the clinical utility of PGx testing shouldn’t be restricted in the drug labelling but it should be properly addressed to have a correct use and application of the PGx testing in practice. For instance, it would be important to promote PGx counseling conducted either by specialized and well-trained clinical geneticists or by clinical pharmacologists who are able to interpret PGx results and give guidance to both physicians and patients. The corresponding professionals may have the expertise whereas they can be certified from accreditation systems to become an important component of the workflow.

However, educating and sensitizing healthcare professionals isn’t enough to ameliorate the adoption rate of PGx as there are many stakeholders involved in the field. To improve the implementation of PGx testing is crucial to properly inform governmental bodies, insurance companies, third-party payers, and of course, patients and the public. Patients are one of the pillars of healthcare systems. As they are the end-user of healthcare services, their opinion and active engagement are key determinants for the future of PGx in many ways. Indeed, public involvement may give a boost to existing scientific research either by funding a greater number of PGx programs or by providing young researchers with grants and fellowships to advance their projects. This fact would most likely enrich the evidence base and in combination with cost-effectiveness analysis, it can bring a change in the attitude of the state and insurance companies, which aren’t presently willing to invest and reimburse PGx testing in many countries. Undoubtedly, there are many ways to engage public engagement either via brochures or a series of TV, online, or on-site campaigns including outreach activities organized in public spaces such as parks, schools, nursing homes to properly inform and motivate individuals of all ages and social groups.

Achieving PGx testing reimbursement seems to be quite challenging but it isn’t impossible while it can bring several changes in society. Such a change could be the amendment of existing legislation regarding genetic data protection to avoid any unauthorized access due to regulatory gaps. Many patients and healthcare professionals have pounded the alarm about the possible misuse of health data and the related issues and therefore it is urgent to proceed with that kind of legal reform. In addition, it would be important to reduce the time required for PGx tests to get approval from regulatory bodies. This step would spark the interest of companies to invest in the development of relevant products, it would promote health innovation and it would decrease the cases of non-accredited suppliers. In parallel, enhancing the role of the pharmacovigilance department of the various national medicines authorities to maintain better and accurate records as far as genome-related adverse drug reactions (Özdemir et al., 2015) is highly recommended to support the whole initiative.

The issue of clinical validity and the existence of non-accredited suppliers is quite complicated, but it is possible to be properly addressed and resolved following a common framework. Each country could set specific standards concerning safety, quality, validity and acceptability of PGx testing and to perform regular audits to corresponding suppliers such as hospitals, clinics, genetic laboratories, providers with the objective to ensure their proper operation.

Overall, the means of promoting, communicating and advertising the existing PGx and personalized medicine services to the interested stakeholders are still poorly developed. Implementing significant improvements and adopting innovative approaches is required to ensure the broader use of these services by the healthcare community for the benefit of the patients.

## Data Availability

The original contributions presented in the study are included in the article/supplementary material, further inquiries can be directed to the corresponding author.
